# Disability Data Collection in a Complex Humanitarian Organisation: Lessons from a Realist Evaluation

**DOI:** 10.3390/ijerph181910334

**Published:** 2021-09-30

**Authors:** Claire F. O’Reilly, Louise Caffrey, Caroline Jagoe

**Affiliations:** 1 School of Linguistic, Speech & Communication Sciences, Trinity College Dublin, D02 PN40 Dublin, Ireland; 2 School of Social Work and Social Policy, Trinity College Dublin, D02 PN40 Dublin, Ireland; louise.caffrey@tcd.ie

**Keywords:** disability, realist evaluation, food security, complex adaptive systems, humanitarian, sustainable development goals

## Abstract

In recent years, global attention to disability inclusion in humanitarian and development contexts, notably comprising disability inclusion within the Sustainable Development Goals, has significantly increased. As a result, UN agencies and programmes are increasingly seeking to understand and increase the extent to which persons with disabilities are accounted for and included in their efforts to provide life-saving assistance. To explore the effects and effectiveness of such measurement, this paper applies a complexity-informed, realist evaluation methodology to a case study of a single measurement intervention. This intervention, ‘A9’, was the first indicator designed to measure the number of persons with disabilities assisted annually by the United Nations World Food Programme (WFP). Realist logic of analysis combined with complexity theory was employed to generate context-mechanism-outcome configurations (CMOC’s) against which primary interviews and secondary data were analysed. We show that within the complexity of the WFP system, the roll-out of the A9 measurement intervention generated delayed, counter-intuitive and unanticipated effects. In turn, path dependency and emergent behaviours meant that the intervention mechanisms of yesterday were destined to become the implementation context of tomorrow. These findings challenge the current reliance on quantitative data within humanitarian-development disability inclusion efforts and contribute to our understanding of how data can best be leveraged to support inclusion in such contexts.

## 1. Introduction

Disability inclusion (DI) is an increasingly prominent concern across humanitarian and development work. The collection, analysis and use of disability-relevant data, particularly disaggregated data, has been championed as a way to operationalise the DI agenda and advance the sustainable development goals (SDG) stated aim of ‘leaving no one behind’ [1,2]. Measurement of disability is not straightforward—multiple measurement approaches exist with choice between them influenced by the purpose of the data collection as well as the underlying ‘model’ of how disability is considered and understood [3]. We conceptualized the implementation of disability data collection as an ‘intervention’, in that it has an underlying theory (implicit or explicit) as to how it can bring about an outcome or change within the UN World Food Programme (WFP) system. While guidance exists on selecting appropriate measures (e.g., Washington Group Modules [4], less attention has been paid to the process of implementing disability-related data collection within complex organisations, and the ripple-effects of such interventions. Understanding how a complex system responds to disability data interventions is crucial if such data interventions are to be mainstreamed across humanitarian and development organisations. 

WFP is the world’s largest humanitarian organisation, created in 1961 to provide multilateral food assistance. Known colloquially as the ‘logistics engine’ of the UN system, WFP has developed an organisational preference for using quantitative data to support operating at scale; measuring billions of funding dollars received, thousands of metric tonnes of food delivered, and tens of millions of persons assisted annually. In the recent context of growing sectoral attention to DI, WFP made several organisational commitments, including disaggregation of its beneficiary data by disability, alongside sex and age. In 2018, this became the trigger for the launch of a new performance indicator—‘A9’—measuring the number of persons with disabilities reached by WFP assistance. A9 was thus designed as an output indicator [5], meaning it collected annual data on the persons assisted by WFP programmes, by measuring the number of persons with disabilities assisted. Therefore, A9 is distinct from disaggregating needs assessment data, which could provide information about whether persons with disabilities are disproportionately represented among households without enough to eat, or outcome indicators, which might measure the extent to which being included in assistance resulted in improved nutrition profiles for this group. Implemented across the complex, adaptive system of WFP itself, and spanning operations across 88 diverse country contexts, A9 launched one year before the UN Secretary General introduced the UN Disability Inclusion Strategy (UNDIS), putting WFP at the forefront of efforts to systematise collection of disability data across life-saving humanitarian and development operations. 

By 2020, WFP recognized that A9 was not functioning as intended and, as part of a partnership with Trinity College Dublin, set out to understand why and what could be done differently as they advanced the disability inclusion agenda. Here, we used a complexity informed, realist methodology to build an empirically testable account of ‘how’ and ‘why’ the A9 indicator resulted in myriad outcomes across WFP’s various operating contexts. What seemed like a small change—disaggregating one indicator among many, had system wide, unanticipated effects. Over the 3-year history of the intervention, the bi-directional dynamics of organisational change meant that the WFP system responded to A9, while the A9 indicator was itself manipulated and revised through its use and interpretation. This layered nature of organisational change meant that as disability measurement expanded, the intervention mechanisms of yesterday were destined to become the implementation context of tomorrow. Our goal is to assess how a disability measurement intervention operated in practice, to help improve understanding of how best data can be leveraged to support inclusion in humanitarian and development activities.

## 2. Materials and Methods

This paper presents a case study, using realist evaluation, of the development and implementation of WFP’s A9 indicator. This case study of A9 forms one component of an ongoing, realist evaluation of the use of evidence as a pathway to mainstreaming disability-inclusive programming in WFP. Realist evaluation is a theory driven, explanatory focused research methodology that seeks to answer the question ‘what works, for whom, in which contexts, and why?’. It does this by identifying underlying, generative mechanisms (M) which, in certain contexts (C), operate to produce observable outcomes (O), framed as CMO-configurations (CMOC) [6]. Mechanisms are not elements of a given intervention—in our case, A9—but rather are a heuristic to represent how programme stakeholders—here, WFP staff—respond to the resources provided by the intervention. Mechanisms and the outcomes they produce are context dependent, as only certain conditions will enable the ‘firing’ of the mechanism [7]. This makes the variety of contexts across which WFP operates rich ground to evaluate a common intervention such as A9.

The aim of a realist evaluation is to identify generative mechanisms that can inform transferable guidance—the end result is knowledge which is partial but cumulative, and which can inform further theorizing [6]. To achieve this, CMOC’s are developed and then refined in an iterative, theory-informed process, whereby the use of retroductive reasoning seeks to reveal the underlying causal explanation for observed outcomes. To ensure rigour, investigators should explicitly outline how the research draws on theory to develop CMOCs, which are then tested against the data. Practically, we drew on elements of complexity theory as applied to organisations. Complexity theory provides a language and conceptual framework for thinking about implementation and change dynamics in complex systems [8,9,10,11,12,13,14]. It therefore provided a lens to frame our theorising and develop CMOC’s which were subsequently refined, augmented, or refuted through analysis of secondary, quantitative A9 data, and qualitative interviews with WFP staff. We used this complexity informed, realist methodology to ask; how did one indicator among a suite of many, become adapted, in such a wide variety of ways, resulting in intended and unanticipated effects? 

The case study unfolded across 3 phases: theory gleaning; generation of initial programme theories (formatted as CMOC’s); and refinement of CMOC’s through analysis of secondary data and primary interview data. 

### 2.1. Theory Gleaning

To begin, we sought to make explicit the underlying theory of A9, to understand by which mechanisms and to what outcome its designers expected the intervention to function. Manzano [15] describes “theory gleaning” as a key initial phase in a realist approach, and here we drew on our embeddedness as researchers linked to WFP on a broader, multi-year partnership, and from C.O’R. and C.J.’s observations as participants in the sector ourselves (humanitarian-researchers). To capture both ongoing and contemporaneous discussions, we participated in calls with the indicator development teams and its users and reviewed internal WFP email threads referring to A9 development and implementation.

This initial phase was key as it revealed many underlying motivations and considerations that might not be recalled in later interviews, thus focusing the direction of data collection toward specific theory refinement. As we proceeded, we understood that at the time of its conceptualisation and launch, key WFP staff viewed A9 as an initial move toward meeting its public commitments on collecting disability-related data. The theory gleaning phase also served a practical purpose, as it fostered relationships with WFP staff that facilitated access to A9 data. It was our experience that building this trust contributed to greater openness in subsequent interviews, giving us deeper insight to stakeholders reasoning regarding A9. This phase confirmed our expectation that A9 could serve as a ‘ready-made’, natural case study as WFP embarked upon standardising the use of disability-related data.

### 2.2. Generation of Initial Programme Theories and Structuring CMOC’s

The theory gleaning phase underpinned the development of initial theories about how and why A9 functioned, and these theories, along with a document analysis informed by complexity theory, contributed to CMOC’s. Realist evaluation has been criticised for a lack of clear detail about how CMOC’s are constructed [16]. In our document analysis, we drew on Gear and colleague’s [17] use of complexity theory as a qualitative research methodology, to analyse the function of the document, rather than the content. That is, rather than analysing what A9 ‘said’, we focused on what it ‘did’ as a resource. Other secondary documents helped to elucidate this function by illustrating the broader discourse in which A9 sat, and therefore what needs it might address. These included records from WFP Executive Board meetings, corporate planning documents, and UN and sectoral guidance documents on disability inclusion and data collection. 

In realist evaluation, middle range theory (MRT) is employed to “explicate the underlying logic of programs” [18] through CMOC’s; theoretical propositions which are then tested against the evidence. MRT lies between day-to-day working hypotheses and “systematic efforts to develop a unified theory that will explain all the observed uniformities of social behaviour, social organization and social change” [19]. We used complexity theory as our MRT, with prominent realists noting that complexity and realist evaluation are “natural bedfellows” [20]. While critiques of complexity theory have described it as more “a descriptive conceptualisation of the backdrop to action” [21], we put complexity theory to work, drawing on it as a framework upon which to build an empirically testable account of ‘why’ A9 worked as it did. MRT allows researchers to move beyond descriptions of regularities, such as ‘X number of country offices reporting on A9 using X data’, to their explication—why was A9 adopted or ignored in such a variety of ways? 

We note that our MRT, complexity theory, is a framework rather than a unified theory, and some components, such as systems theory, may have ambitions to grander monikers than mere ‘middle’ range theory. However, complexity comprises and delineates multiple useful concepts, including “open” systems, path dependence, feedback loops, emergence and self-organisation, which informed the development of CMOC’s, to which the interviews were coded. Table 1 below provides a brief definition of each concept used in this study.

### 2.3. Analysis of Secondary Data and Interviews

The initial CMOC’s were refined, augmented or refuted through analysis of secondary A9 data and qualitative interviews with WFP staff. We analysed the sources of the data reported for A9, such as surveys, estimates, etc., as these sources indicated how WFP was using and responding to A9 in practice. Drawing on the realist concept of depth ontology, we focused not on the numbers produced by A9, but at a deeper level, analysed the underlying sources for these numbers, and what this indicated about how A9 was being used (Table 2). Drawing on aggregated annual country reporting from 88 country offices we analysed whether (a) data pertaining to A9 was reported and if so, (b) the source of the data (e.g., monitoring surveys conducted by the country office itself, secondary data generated by other actors; the accepted global prevalence estimate of 15%, etc.) [22]. 

A total of 19 participant interviews were conducted as part of the larger realist evaluation of implementation of disability-related data collection efforts in WFP. A subset of these interviews (n = 9) either made unprompted mention of A9 or were iteratively adapted to include questions related to A9. All interviews were conducted remotely and were consistent with the ‘teacher-learner’ cycle of realist interviews, whereby theories are explicitly shared with the interviewee for comment [15]. The participants included WFP staff in a range of different roles, including protection officers, data specialists, programme managers, and specialist thematic advisors. Interviewees also spanned specific country offices, regional bureaux and global, HQ level staff, providing insight into how different levels of the organization was responding to A9. All interviews were conducted by the lead author and used a semi-structured guide to lead the interview process. All interviews were recorded and transcribed verbatim, and coding was carried out using NVivo V1.5. [23]. The CMOC’s presented below were developed during the theory gleaning phase and were used as the unit of analysis, whilst themselves being iteratively refined through this process. The Research Ethics Committee of the School of Linguistic, Speech & Communication Sciences at Trinity College Dublin granted ethical approval for the study.

## 3. Results

**Figure 1:**
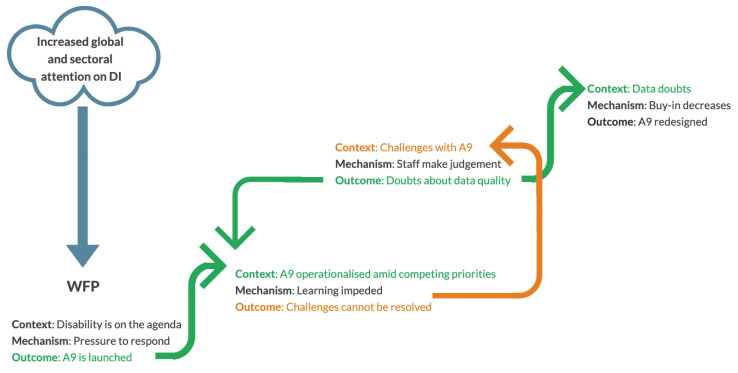
Stackable CMOC’s: Outcome Becomes Context.

In this section we present the CMOCs in a ‘stackable’ order (Figure 1), whereby one outcome can be seen to influence the context in which subsequent mechanisms may operate. This illustrates the complexity concept of ‘path dependency’, whereby the history of the intervention or system influences its current and future expression and state. Each CMOC is presented alongside demonstrative quotations which illustrate our theoretical propositions about how the complexity of the WFP system influenced how and why A9 worked, for whom, and in which contexts.

### 3.1. Disability Is on the Agenda: Fluid Boundaries in a Complex System

CMO-configuration: In the post-2015, SDG agenda, DI was a new priority among humanitarian and development actors and the donor governments who fund WFP’s work, but it was not yet a systematised element of WFP practice (context). As a standard setting, voluntarily funded organisation, WFP perceived pressure to respond (mechanism) to the growing DI imperative with demonstrable action. This led WFP staff to design and launch the novel A9 indicator, making it mandatory for all country offices to measure and report the numbers of persons with disabilities receiving WFP assistance (outcome).

These influences were evident in minutes from WFP’s Executive Board meetings, made up of donor government representatives, where enhanced reporting on the inclusion of persons with disabilities in WFP activities was repeatedly requested. Contemporaneously, relevant donors and other UN agencies and humanitarian and development actors were publishing materials highlighting an increased focus on disability inclusion. Within this context, A9 likely served dual political and practical purposes; demonstrating to donors that WFP was taking DI seriously, and directing field staff on how, where, and when to start collecting data disaggregated by disability.

The following quotations, taken from the primary interviews with WFP staff, illustrate their response to disability-related data as it was emerging within the WFP landscape, with a sense of permeability between actors in the broader humanitarian-development context:

**Monitoring & Evaluation (M&E) Staff:** “*I mean, we can see it everywhere, not in WFP, but we can see it in the world news in everything—that people with disabilities now is on the agenda. I don’t know how they got there, but they are in the agenda, right*?”

**Protection Staff:** “*I think it had been raised a lot within the executive board, with the member states. I don’t know exactly why, but for sure, there was, it had been raised by a few members*.”

Across the humanitarian-development sphere, WFP is embedded in multiple systems where boundaries and, in-keeping with complex systems, “the notions of ‘inside’ and ‘outside’ are never simple or uncontested” [24]. For example, WFP publicly committed to sectoral initiatives to mainstream DI, including disaggregation of data, and through its involvement with the wider UN system WFP is accountable to the UN Disability Inclusion Strategy. Meanwhile, the donor governments who make up WFP’s Executive Board were themselves subject to realizing their own commitments to DI, the majority having ratified the Convention on the Rights of Persons with Disabilities.

Complex, open systems are characterized by ‘fuzzy boundaries’, where boundaries constitute both the activity of the system, and the way in which those boundaries are described. WFP considers itself a food security organisation, which responds and prioritises assistance based on need. Disability, therefore, could be perceived as shifting its modus operandi to privilege a person’s ‘status’, i.e., whether or not they are identified by WFP as having a disability. However, given the malleability inherent in complex adaptive systems, and at least partly in response to interpenetration with surrounding systems, the creation of A9 moulded the boundaries of WFPs mandate to consider more explicitly, the relationship between disability and food security needs. 

### 3.2. We’ll Call You back: Frustrated Organisational Learning

CMO-configuration: WFP designed and launched the novel A9 indicator, making reporting mandatory for all country offices (CO’s) globally, but competing workstreams and priorities across WFP’s high-need, limited-resource operational environment (context), meant that learning was impeded (mechanism), with the result that although WFP staff identified issues implementing A9, these could not be adequately addressed (outcome).

From our earlier theory gleaning, including review of contemporaneous internal email threads referring to A9 development and implementation, we surmised that A9 was designed as an adaptation of the Washington Group Short Set of Questions. This is an internationally validated tool for disaggregating data by disability and is increasingly favoured by sectoral actors and donors [4,25]. The adaptation was designed to simplify use, and A9 was designated an ‘output’ indicator, meaning it was applied only to a subset of households that had already received WFP assistance. Ultimately, these decisions affected both the process and substance of A9 is such a way that the indicator struggled to provide data that was of reliable quality, and at a point in the programme cycle where the data could provide information to refine and inform implementation:

**M&E Staff:** “*The intention was in any case to start gathering some data, make COs aware of the need to think about disabilities, and to gather lessons learnt and improve. So, it was a first step…We wanted to profit [from the opportunity] to include and to start, you know? And then after that, I did go back to the technical unit a couple of times to say, “Guys, I mean this is the information we have gotten—it’s not very good. These are the lessons learned. So it’s time to revise these methodologies.”…they told me, “OK, wait a little bit. We’ll call you back when we are able to touch on this topic*.”

Although WFP staff viewed A9 as an initial move toward meeting its organisational commitments on collecting disability-related data, feedback loops; the mechanism by which experiential learning could have refined A9 through its life cycle, were initially frustrated due to the realities of WFP’s competing priorities and constrained resources. A negative (balancing) feedback loop is a learning loop which enables corrective action when departures from intended actions or errors occur. Although A9 underwent at least one revision in the three years of its operation, as evidenced in WFP’s indicator compendium, there was insufficient feedback and corrective action to address the underlying implementation challenges. This inadvertently created a ‘cul-de-sac’ whereby implementing A9 could stand as a self-contained outcome, i.e., data for data’s sake, rather than as an informative step toward inclusive action. Thus, A9 was functioning as an end rather than a means toward more inclusive programming. This resulted in confusion and frustration among staff who expected that disability disaggregated data should contribute to planning programmes or understanding the inclusion gap, but found A9 struggling to do either.

### 3.3. This Is Really outside the Box: Emergent Behaviour in the Face of Data-Doubts

CMO-configuration: In the face of WFP staff experiencing issues implementing A9 and with initially limited internal expertise to draw on support implementation, A9 continued to be mandatory, with donors and senior management requesting the data (context). WFP staff at various levels began to judge (mechanism), on a case-by-case basis, whether and how to report on A9. Consequently, different country office’s variously applied or ignored A9, with the result that at an organisational-level, A9 did not reliably produce useful and comparable data (outcome).

A9 was a ‘thin’ resource, in that it was WFP’s first corporate measurement approach for DI and so for a period stood alone, tied to neither training or wider guidance. The resultant response, as A9 was adopted in advance of dedicated work to embed and reinforce DI across the system, was correspondingly scanty in terms of reliable data. The following quotes illustrate the concerns related to the validity of the data and the need for support:

**Regional Support Staff:** “*But then as I as I just mentioned, in most cases, that numbers just seem so ridiculously low that I would rather remove it, although it’s mandatory to report on. But I would rather remove it or just use the 15 percent overall number sort of as a blanket thing...* *the numbers that the country officers were reporting was so much lower [than the global average] that it almost seemed really ridiculous to report it*.”

**Programme Manager Staff:** “*if you’re going to be introducing anything that’s new, and this [A9] is going to be really out of the box for pretty much everybody, it cannot come without guidance methodology. How do we really collect this? What is the relevance of it? How do we analyze and interpret it and apply it? So that, I think, has to be there*.”

Agents within a system are themselves complex, giving rise to the system’s capacity to innovate on the ground and overcome structural gaps [26]. Despite challenges, by its second year of implementation the majority of WFP country offices (CO’s) had found ways to report on A9. The secondary data analysis gives an indication of the emergent behaviour in response to A9, demonstrating a variety of reporting sources and interpretations. Some of this variety was expected and encouraged by the A9 methodology itself, which detailed various reporting options. Unanticipated however, was the way in which applying general disability prevalence data to WFP beneficiary tallies implied the proportionate inclusion of this group and would thus have obscured both higher representation due to increased food insecurity and need, or decreased representation, due to difficulties for WFP to identify such households, or for them to access WFP assistance.

Table 1 shows the number of WFP CO’s reporting on A9, and the total number of beneficiaries with disabilities identified. These reporting sources provide insight to how the users, WFP staff, were responding to and taking up the resource of A9.

### 3.4. It Doesn’t Give Me Any Information: Self-Organisation to Get the Job Done

CMO-configuration: WFP staff were increasingly wary of the data being produced by A9, and they struggled to use the data to inform the design or application of assistance programmes to ensure the inclusion of persons with disabilities (context). Over time, buy-in and support for A9 decreased as doubts increased (mechanism), reducing A9′s credibility and sustainability, triggering a re-design of the indicator (outcome).

The authors of A9 envisioned it as a necessary first step on the path toward DI, and may have hoped to harness the complex adaptiveness of WFP by planting a first seed which could then grow awareness through the organisation. However, causal processes are not linear, and unpredictability as to where a first step may lead is compounded by self-organisation within the system. Given agent’s ability to organize and respond to change, a new intervention can be “defeated by the systems response to the policy itself” [4], as illustrated by the participant quote below:

**Protection Staff Member:** “*We didn’t at that stage understand why we were collecting the data. So we collect data because we were required to collect the data. But the data wasn’t being used to inform programming. And that’s very obvious in the fact that, you know, disability was considered an output indicator and not an outcome indicator. And I need it to be an outcome indicator to be able to directly correlate it with how we adjust programming. As an output indicator it provides me with very little information beyond: this is how many people with disabilities we provided assistance to. But that doesn’t give me any information on whether they are facing challenges, whether we needed to establish new measures for them*...”

Agents in this (social) system had flexibility and agency in how they interpreted and responded to the resource of A9. Their response to the resource—in realist terms, the generative mechanism—was influenced by their context, which in turn was influenced by the outcomes of previous context-mechanism interactions. As we have seen, these included inadequate pathways to refine A9 through use, and A9′s inability to produce reliable data at a point in WFP’s programme cycle that could influence change. This resulted in decreasing buy-in for A9, ultimately culminating in a review and redesign of the indicator, of which this analysis was part.

## 4. Discussion

The case study of WFP’s A9 indicator allowed us to map “the contours of complexity” at the outset of this multi-year evaluation [27], and to account for the causal pathways of outcomes from a disability-related measurement intervention. Our review demonstrates that getting any disability inclusion measurement ‘just right’ may not be enough to support the outcome of increased disability inclusion, if insufficient attention is given to guiding how the resource will be utilised in practice to generate outcomes. Using CMOC’s, we purposely abstracted to the range of MRT, using complexity as a theoretical framework to package and explore the linkages between contexts and mechanisms. We cannot claim that the CMOC’s we elucidated capture all the pertinent, mechanistic elements of implementing data disaggregation across a complex organisation. However, amidst ongoing high enthusiasm for disability-disaggregated data from donors and practitioners alike, these configurations can be picked up by other practitioners as a ‘portable theory’ to inform their own efforts. These findings are relevant not just to understand A9, but to highlight the potential pitfalls to be avoided in the implementation of disability inclusion and its measurement across humanitarian and development work.

While the production of organisation-wide, comparable data may have been a reasonable aim of A9, uniformity of application could not be achieved, in part due to the impact of the intervention taking place in multiple social realities, across varied contexts. This was recognised by the indicator document itself, which laid out alternative reporting methods, but even within this ‘sanctioned’ variation, unanticipated outcomes arose. The most interesting element here is not the numbers of persons with disabilities reported by A9. Indeed, our first engagement with A9 was in relation to the methodological improvements WFP wished to implement to increase the reliability of the data. Rather, this paper sought to understand how the intervention—regardless of its inherent quality—was operationalised across the system, as “process data provides a useful explanation of the observed outcome” [27].

The answer to ‘what works’ is always ‘it depends’, with the answer dependent upon for whom the intervention is intended to work, and in which context(s) [28]. To ascertain whether A9 ‘worked’, it was necessary to first elucidate exactly what A9 was trying to achieve; that is, its underlying theory. Although interventions seek to address problems, themselves manifestations of causal, generative mechanisms, we found a lack of clarity as to the problem A9 was designed to address.

When A9 was launched, disability inclusion was seen as a problem to be solved, rather than a system state to be achieved. A9 was introduced at a time when the humanitarian-development context was in flux in terms of DI, and the characteristically short timelines of both funding and implementation across the sector compelled staff to react quickly. This enabled a reductionist approach whereby a linear solution—to count the number of persons with disabilities reached annually, using a simplified, untested adaptation of the Washington Group Questions, was insufficient to achieve the system-state change of inclusivity toward which sector-wide DI efforts ultimately strive. Given that “a critical feature of all programmes is that, as they are delivered, they are embedded in social systems” [28], the success of any intervention is reliant not only upon the merit of that original idea, but also upon the context in which it is implemented. A9 was a means that existed before an ends—it measured one aspect of disability inclusion, before WFP had systematically articulated its intention to become a disability inclusive organization, and thus why the inclusion of persons with disabilities was useful to measure.

Through its communication and interpenetration with other complex, open systems, including the wider multi-lateral system of donor infrastructure and the humanitarian-development sector, WFP developed and launched its goal of becoming a disability-inclusive organisation writ large. Since 2018, when A9 was conceptualised and launched, WFP has expanded its engagement with DI and by 2020, WFP had introduced a new implementation roadmap, alongside dedicated human resources and an increasing number of supportive—and demanding—donors. As the context became more favorable to DI overall, the outcomes produced by A9 were increasingly unsatisfactory. A9 did produce data for the majority of WFP’s country contexts over multiple years, but over time, counting the number of programme beneficiaries with disabilities was perceived as an insufficient measurement. As this context around A9 shifted, staff responded to and utilisied A9 in multiple ways, but ultimately agents in the system could not overcome the lack of a pathway for A9 data to inform WFP activities through beneficiary targeting or programming design.

To avoid humanitarian data collection earning the dreaded label of being a ‘tick-box’ exercise, thought must be given to the type and purpose of data collected, with careful planning to ensure the information generated has a pathway to inform action. We acknowledge that while complex adaptive systems are difficult if not impossible to predict, it may still be possible to guide sub-components of the system toward common outcomes, provided a unified vision of the ultimate goal is clearly articulated and disseminated. This evaluation of A9 showed that until the resource of A9 is sufficiently ‘thickened’ or augmented by additional measurement and support, it is likely that CO’s will continue to manipulate the indicator and struggle to action the data in their activities.

The history of an intervention cannot be overlooked, as it shapes the context, which is key to success or failure [27]. As WFP continues strengthening efforts towards and measurement of DI, consideration of the lessons learned regarding constraints and characteristics of complex systems operating within a political, results-contingent context is necessary to ensure change that is effective and sustainable. A balancing strategy such as a functional feedback loop to enable organisational learning (i.e., A9 is formally refined as lessons are learned through implementation) was required to disrupt unwanted and unintended outcomes, and this ultimately occurred in part through the commissioning of this research.

This case study has limitations. As is common for a humanitarian organisation, and is exacerbated during a global pandemic, staff turnover was high as people were redeployed to new emergencies or assumed new roles. This meant that it was not possible to identify or reach all persons involved in the conceptualisation of A9; while resource constraints and practicality made interviewing all staff who interacted with A9 unfeasible. This may have created bias in the interview sample and excluded important perspectives from informing and refining our theorising. Due to travel restrictions imposed by COVID-19, interviews were conducted by phone or via online audio/video platforms, however we propose that this did not significantly impact data quality, as given the nature of their work, WFP staff were already habituated to working remotely.

## 5. Conclusions

Social change has a bi-directional nature, as people’s actions and agency are shaped by community and institutions, and these structures change over time as a result of who comprises them [29]. Successful interventions adapt over time to respond to evolving needs and integrate with structural levels above and below to produce outcomes. To accommodate this evolution, systems should be flexible enough to allow for innovation to subvert, rearrange or eliminate elements that are obsolete. While A9 was a first step, as WFP continued down the path of DI, the organization increasingly realized that from a measurement and monitoring perspective, more was needed.

Ultimately, the head-counting of disaggregation alone presents too reductive a solution to the complexity inherent in large humanitarian-development organisations, their operational contexts, and the problem of disability exclusion itself. To achieve the system state of ‘disability inclusivity’, data-disaggregation alone will be insufficient. The CMOC’s presented here have explanatory power as to how and why disaggregation can produce outcomes other than those intended, pointing to the need for the sector to look beyond only quantitative disaggregation as the evidence base for inclusive action, and to anticipate complexity wherever such measurement is implemented.

## Figures and Tables

**Table 1:** Complexity Theory Concepts.

Concept	Definition
Fuzzy Boundaries	Unlike mechanical systems, for example, a space rocket, which have a clear boundary, complex adaptive systems are open and so the boundaries are fuzzy. Agents and activities in the system may be part of several other systems and membership of the systems is not fixed but evolving.
Path Dependence	The outcome of a process depends, not simply on current circumstances but on past history—on a sequence of decisions made by agents and resulting outcomes. The trajectory of a system is limited by path dependence but is not wholly determined by it.
Organisational Learning	Organisational learning is the process by which organisations change or modify their mental models, rules, processes or knowledge to affect their performance. Learning involves the detection and correction of error. A feedback loop is a recursive mechanism that creates behaviours that reverberate back on themselves. A positive (reinforcing) feedback loop increases the rate of change, reinforcing its own output. When it is undesirable, it creates a ‘vicious circle’. A negative (balancing) feedback loop is a learning loop. It monitors what is happening, detects errors and departures from goals, and implements corrective action.
Emergence	Interactions between the parts of a system can produce ‘emergent’ properties that cannot be understood by examining each part in isolation.
Self-Organisation	The way in which agents in a complex system organize themselves in the absence of or despite external control, direction, pressure or influence, creating constant, dynamic system evolution.

**Table 2:** Data sources for A9 reporting.

Source	No. of Country Offices	
	2019	2020
WFP Monitoring Survey	15	20
WHO Global Disability Prevalence Estimate (15%)	1	1
Secondary/Partner Data (Various Sources)	33	7
UNHCR ^1^ Registration Statistics	3	2
National Statistics/Census	8	3
Beneficiary Registration (Medical Diagnosis Questions)	3	7
Combination (e.g., census and UNHCR data)	3	5
Estimate	4	1
Other (“simple counting”, “head count”)	4	1
Total no. of beneficiaries with disabilities identified	3%	6%

^1^ United Nations High Commissioner for Refugees.

## Data Availability

3rd Party Data Restrictions apply to the availability of the secondary data presented here. Secondary data were obtained from WFP and are available from WFP with their permission. Primary qualitative data presented here are not publicly available due to the ongoing nature of the larger research project of which this study formed part, meaning that participant confidentiality could be compromised.
